# Personal Factors, Internet Characteristics, and Environmental Factors Contributing to Adolescent Internet Addiction: A Public Health Perspective

**DOI:** 10.3390/ijerph16234635

**Published:** 2019-11-21

**Authors:** Sulki Chung, Jaekyoung Lee, Hae Kook Lee

**Affiliations:** 1 Department of Social Welfare, Chung-Ang University, Seoul 06974, Korea; 2 Korea Center on Gambling Problems, Research and Development Team, Seoul 04554, Korea; jklee@kcgp.or.kr; 3 Uijeongbu St. Mary’s Hospital, The Catholic University of Korea, Uijeongbu-si 11765, Korea; nplhk@catholic.ac.kr

**Keywords:** Internet addiction, public health model, Internet game advertising, accessibility, environmental factors

## Abstract

Individual characteristics, family- and school-related variables, and environmental variables have equal importance in understanding Internet addiction. Most previous studies on Internet addiction have focused on individual factors; those that considered environmental influence typically only examined the proximal environment. Effective prevention and intervention of Internet addiction require a framework that integrates individual- and environmental-level factors. This study examined the relationships between personal factors, family/school factors, perceived Internet characteristics, and environmental variables as they contribute to Internet addiction among adolescents based on the public health model. A representative sample of 1628 junior high school students from 56 regions in Seoul and Gyeonggi-do participated in the study via questionnaires with the cooperation of the Ministry of Health and Welfare and the district office of education. The study analyzed psychological factors, family cohesion, attitudes toward academic activities, Internet characteristics, accessibility to PC cafés, and exposure to Internet game advertising. About 6% of the adolescents were categorized as being in the severely addicted group. Between-group comparisons showed that the addicted group had started using the Internet earlier; had higher levels of depression, compulsivity, and aggressiveness as well as lower family cohesion; and reported higher accessibility to PC cafés and exposure to Internet game advertising. Multiple logistic regression indicated that for adolescents, environmental factors had a greater influence than family or school-related factors. Policy implications for prevention and intervention are discussed.

## 1. Introduction

As Internet usage has rapidly increased, it has become a part of our lives and is both a medium for providing a wealth of information and an important tool for connecting with others around the globe [1]. The advent of smartphones and easy access to the Internet have brought about many advantages. However, many people have developed concerns about such ease of access and social networking, particularly for adolescents; one concern is Internet addiction. Although not a formal diagnosis, Internet addiction is becoming widely accepted as a problem that may require professional treatment [2]. There is a lack of consensus regarding the definition of Internet addiction, and the term has been used in different studies with different connotations. In general, Internet addiction is defined as excessive use of the Internet accompanied by withdrawal, tolerance, and negative repercussions [3]. For consistency reasons, we utilize the term Internet addiction throughout this paper to represent a pathological state that occurs due to Internet overuse. A school-based survey for 9th and 10th grade students in seven European countries (Greece, Spain, Poland, Germany, Romania, the Netherlands, and Iceland) revealed that 0.8% to 1.7% of the students were suspected to have Internet addiction using Young’s Internet addiction test [4]. A meta-analysis that reviewed studies from 31 nations reported that the global occurrence of Internet addiction was estimated at 6% [5]. However, higher estimates of the problem have been reported in Korea. Reportedly, almost 100% of South Korean adolescents are Internet users [6], and Internet addiction is a serious public health issue among Korean adolescents [7,8]. According to a national survey, adolescents had the highest proportion of high-risk Internet dependence (13.1%), followed by adults (5.8%), and then children (5.0%) [9]. Survey results from different countries also reported that adolescents as a group are the most vulnerable to Internet addiction [10]. Children and adolescents, having grown up in the digital technology era, have been familiar with digital devices and the Internet from an early age. A typical 15-year-old in 2015 would have been using the Internet since age 10 and the hours spent on the Internet is noticeably increasing among Organization for Economic Co-operation and Development countries [11]. Research studies in addiction have indicated that exposure to substance use at an earlier age increases the risk for addiction and risky behaviors later in life [12,13]. This can also be said for Internet use at an early age, and there is a concern that early access to electronic devices could lead to escalated risks for addiction [14]. Also of importance is the contents of the Internet. Different contents of the Internet affect young people to engage in overuse of the Internet. For instance, Greenfield [15] suggests that the most addictive aspects of the Internet are sexual content and computer gaming. Although these content areas are not limited to the Internet, when these are accessed using the Internet, the addictive potential of the contents are known to be amplified. A recent study also reported that people who frequently engage in web surfing experience the same psychological effects as those who are hooked on gambling [16].

Mental health experts believe that Internet addiction may manifest the same troubling effects as substance abuse or gambling disorders [17]. Internet addiction has been associated with various physical and psychological problems, and Internet addiction mostly manifests in adolescents as social withdrawal, loneliness, low motivation, and low educational performance [18].

Many studies have been conducted to identify the factors associated with Internet addiction. Of these, a number of studies have documented personal risk factors, typically psychological factors. These include depression [18,19,20,21], anxiety [20,21], aggression [22], impulsivity [23], and low self-esteem [21,24]. Among many personal risk factors of Internet addiction, depression, aggression, and impulsivity are of particular interest due to their association with other clinical subtypes. Depression is related to emotional vulnerability or internalizing problems, and aggression and impulsivity are related to externalizing problems such as attention deficit hyperactivity disorder, which are both known to increase the risk for Internet addiction [25]. There is strong evidence that individuals with higher levels of depression are more vulnerable to developing Internet addiction [19,26,27,28,29]. A recent longitudinal study also confirmed that depression is a positive predictor of adolescent Internet addiction [30]. Some studies have identified aggression as a predictor of Internet addiction. In a cross-sectional study of Lebanese adolescents, higher levels of aggression were associated with a higher level of Internet addiction [27]. A similar pattern was witnessed for young people in Taiwan and Korea [22,31]. Impulsivity is another psychological trait that is positively associated with Internet addiction. Several studies have discussed the correlation between high impulsivity and Internet addiction among adolescents [32,33], and a similar finding was reported among young people with internet gaming disorder [34].

Some studies addressed the extent to which interpersonal variables contribute to Internet addiction. They sought to explain Internet addiction in terms of interpersonal difficulties, and confirmed the role of family- and school-related variables in relation to Internet addiction among adolescents. For instance, conflict with parents, family functioning, and family resilience have been identified to influence Internet addiction [35,36,37]. School-related variables such as teacher’s support and attitude toward school life are also associated with young people’s Internet addiction. This is especially true for Korean adolescents, where there is enormous pressure for high academic achievement [38].

Another set of factors that has received relatively less attention is related to the characteristics of the Internet and its influence on addictive behavior. Few studies explicate Internet addiction by understanding what factors of the Internet enhance or reduce a user’s excessive form of use. Several researchers have discussed the seductive and gratifying properties specific to the Internet that attract users [39,40,41]. They claim that certain properties of the Internet are associated with an elevated risk for Internet addiction. For instance, Turkle [42] isolated two dimensions of the Internet that are seductive to the Net generation: the pleasure of control and the perceived fluidity of identity [41]. Several other seductive factors were identified such as self-presentation, diversion, relationship building, and virtual community to name a few [40,41].

Most existing literature on Internet addiction has focused on individual and psychological factors and the proximal environment such as family and peer groups. Extensive meta-analysis or systematic review studies on risk factors of Internet addiction focused mainly on intrapersonal or interpersonal variables [10,31,43], and social environmental factors have received relatively less attention. The studies that have explored environmental factors mostly focused on the proximal environment such as family- and school-related variables [36,38,44]. Only a few studies examined social factors or larger environmental variables (such as exposure to advertisements and accessibility) as they contribute to Internet addiction among young people [45,46,47]. For instance, in alcohol-related research, factors such as availability of alcohol and exposure to alcohol are known environmental factors that influence young people’s drinking behavior [48,49]. It is not difficult to assume that availability and accessibility to the Internet may also affect Internet addiction. However, such factors were infrequently explored in Internet addiction research [46]. Internet addiction is a broader concept that encompasses Internet game addiction. Although this study focuses on Internet addiction in general, we believe that examining the influence of Internet game advertisements as an environmental factor is legitimate given that a large part of Korean adolescents’ Internet use is allocated to playing online games [9].

As discussed above, personal variables, family- and school-related variables, characteristics of the Internet, and environmental variables all contribute to the risk of Internet addiction. Effective prevention and intervention of Internet addiction requires a framework that integrates individual- and environmental-level factors. This is well reflected in the public health model or the epidemiologic triangle based on the public health perspective. Originally designed as a model for infectious disease, the public health model considers three areas of causes in understanding and intervening with any health problems [50,51]. One is the external agent that may contain certain destructive potential. Second is the host, who has individual susceptibility. The last factor is the environment that promotes or discourages certain health-related behavior. Applying the model to Internet addiction, the model focuses on the importance of the host (individual characteristics), agent (characteristics of the Internet), and the environment (i.e., social influence and accessibility) in the manifestation of Internet addiction. The public health model posits that any health problem is a result of the interactions among the three factors. Therefore, the development of effective public health measures for prevention and intervention usually requires assessment of all three components and their interactions [51]. To date, few studies have examined Internet addiction from a public health perspective. An awareness of the influence of these three factors may have a significant impact on intervention, prevention, and policy efforts.

The aim of the current study is to fill the gap in addiction literature by examining Internet addiction among adolescents based on a public health framework. Specifically, the study will explore the relationship among individual variables, family/school variables, the Internet characteristics, and environmental factors as they contribute to Internet addiction. The goal is to identify factors that are more influential when all areas are considered within the framework. To achieve this goal, the study utilizes the Internet addiction test (IAT) developed by Young [52,53]. The IAT has been used extensively in many studies to assess Internet addiction in both the clinical and research fields [54]. It has also been used to identify risk factors [31,43]. However, it should be noted that the IAT is not a diagnostic tool and that categorizing addiction based on the IAT does not define a clinical diagnosis.

The following research questions will be addressed using a representative community sample: (1) Are there any differences between the addict and the non-addict group in regard to psychological factors, family- and school-related factors, perceived Internet characteristics, and environmental factors? (2) Which factors that reflect the agent, host, and environment in a public health model are more important than others in predicting Internet addiction?

## 2. Materials and Methods

### 2.1. Participants

This study utilized a community-based cross-sectional survey. The survey subjects were junior-high-school students who reported the highest risk of Internet addiction in Korea [9]. A representative sample of 1871 middle (junior-high) school students participated in the study. We randomly selected one school from each of the 56 regions in Seoul and Gyeonggi-do that represented both urban and rural districts. Coeducational schools were selected to ensure equal allocation of genders. All students in one randomly selected classroom from each school were asked to participate in the survey between 1 December and 27 December 2015. Questionnaires were mailed to schools with cooperation from the Ministry of Health and Welfare and the district office of education. The study received approval from the Institutional Review Board. Only students who signed the informed consent participated in the survey. Of the 1871 students who responded to the questionnaire, 1628 were included in the analysis after excluding 243 that had at least one missing value in demographic and Internet use behavior variables. Of the 1628 participants, 52.0% (*n* = 847) were male and 47.0% (*n* = 781) were female, and the mean age was 14.9 (*SD* = 0.34).

### 2.2. Measures

#### 2.2.1. Internet Addiction

Young’s 20-item Internet addiction test was used to measure Internet addiction [52]. Each item was measured on a five-point Likert scale. Responses for each item were added to obtain a final score. Many years after the initial development of the IAT, Young proposed a new cut-off point [53]. People who score more than 50 on the IAT are thought to be experiencing frequent problems because of Internet use. For those who score above 80, Internet use is thought to be causing significant problems with their lives. Because the purpose of this study is to examine risk factors associated with the higher risk of Internet addiction, we used the addiction category based on the IAT rather than the overall score. Young proposed four groups of Internet addiction: normal range: 0–30; mild: 31–49; moderate: 50–79; and severe: 80–100. In order to focus on more severe cases, the normal range and mild addiction (low risk) group were combined into one group. Therefore, respondents were categorized into three groups: normal use: 0–49; moderate addiction: 50–79; and severe addiction: 80–100. The IAT in this study held a good internal consistency with a Cronbach’s alpha (∝) of 0.94.

#### 2.2.2. Psychological Variables

The tool used in the study was developed and validated by the National Information Society Agency to identify Internet addiction and coexisting disorders in a national survey on Internet addiction [55]. Each scale consisted of eight questions measured on a four-point Likert scale ranging from one (not at all) to four (very often). Scores were summed for each of the three features with a higher score reflecting a greater degree of impulsivity (e.g., “act on spur of the moment”), depression (e.g., “feeling hopeless”), and aggressiveness (e.g., “have trouble controlling temper”). All three measures possessed good internal consistency (the Cronbach’s alpha was 0.86 for impulsivity, 0.91 for depression, and 0.91 for aggressiveness).

#### 2.2.3. Family and School Factors

The Family Cohesion and Adaptability Evaluation Scales (FACES III) was used to measure family relationship. This scale was originally developed by Olson and colleagues [56], and a Korean version of the scale [57] was utilized in the current study. The family cohesion scale in the FACES III consists of ten questions on a five-point Likert scale that measure emotional ties, family support, time spent together, and interest in leisure time with family. Higher scores reflect higher family cohesion, and the Cronbach’s alpha (∝) was 0.96.

A school adaptation scale developed for Korean students [58] was employed to examine school-related factors. The scale evaluates the degree of adaptation to the school environment, which includes questions about the relationship with teachers, the relationship with schoolmates, attitude toward academic activities, and school participation. The tool consists of four subscales and 14 questions on a five-point Likert scale. Higher total scores indicate a positive assessment of school life. The Cronbach’s alpha (∝) for the subscales were acceptable: 0.94 for relationship with teachers, 0.90 for relationship with schoolmates, 0.62 for attitude toward academic activities, and 0.61 for school participation.

#### 2.2.4. Perceived Internet Characteristics

The agent characteristics in the public health model refer to the traits of the medium in relation to a given problem. In this study, Internet characteristics are defined as the properties of the Internet perceived by individuals in relation to Internet use. Perceived Internet characteristics were measured by the Internet characteristics scale developed by Chung et al. [59] that was designed to identify the characteristics of the Internet perceived by adolescents. It consists of eight factors with 27 items including entertainment, interpersonal relationship, accessibility, profitability, anonymity, immersion, competition, and escape. Based on a previous study that identified three factors to predict young people’s Internet addiction [59], our study included three factors as perceived Internet characteristics: namely, interpersonal relationship (four items; e.g., “the Internet provides opportunities to meet with diverse people”), anonymity (four items; e.g., “People can attract attention without revealing themselves”), and entertainment/pleasure (five items; e.g., “the Internet provides entertainment”). Each item was measured using a five-point Likert scale, from strongly disagree to strongly agree. The interpersonal relationship characteristic of the Internet refers to the perception of the Internet as grounds for building relationships; anonymity refers to the perception of the Internet as a site where people can act freely without identifying themselves; entertainment refers to the perception that the Internet provides fun and excitement. The measures for interpersonal relationships, anonymity, and entertainment showed good internal consistency (∝ = 0.85, 0.86, and 0.87, respectively).

#### 2.2.5. Environmental Factors

Social environmental factors consisted of accessibility to PC cafés and exposure to Internet game advertisements. Previous studies have identified access to PC cafés as a risk factor for Internet addiction among Korean adolescents [47]. PC cafés, a unique feature in Korea, are well-equipped with advanced computers and are optimized for playing Internet games, which attracts many young people. We measured the accessibility to PC cafés using the scale reconstructed by Nam and Lee [60] that consists of a three-item (i.e., There is a nearby PC café for Internet use; I have easy access to PC cafés; there are many PC cafés near my home or school) five-point Likert scale. A higher total score indicated higher accessibility to PC cafés. The Cronbach’s alpha (∝) was 0.85.

We also included exposure to Internet game advertisements as a major environmental factor. Exposure to Internet game advertisements consists of exposure and acceptability. To our understanding, there is no known instrument with which to measure exposure to Internet game advertisements. We used the tool that examined exposure to alcohol advertisements [61,62], except we restructured it to fit Internet game advertisement exposure. Two questions about respondent’s exposure to Internet game advertisements (i.e., How often do you see Internet game advertisements? In the past month, how often did you see Internet game advertisements in the medium that you frequently use?) and the frequency of exposure were measured on a four-point Likert scale. The Cronbach’s alpha (∝) was 0.88.

The advertisement acceptability questions used to determine alcohol use among youths were adopted for the current study [62,63]. However, for the purposes of our study, the questions were modified to assess the acceptability of Internet game advertisements. Two questions were asked: the number of game ads they had seen in various media over the past month and the number of memorable ads that they remember.

### 2.3. Statistical Analysis

To verify the differences between sociodemographic characteristics according to Internet use, a chi-square test (*χ*^2^) was performed. Between-group comparisons of the variables based on the public health model according to the presence of Internet addiction were analyzed using analysis of variance. A logistic regression analysis was utilized to identify the factors associated with Internet addiction. All statistical analyses were performed using SPSS version 20.0 (IBM Corp. Released, Armonk, NY, USA).

## 3. Results

### 3.1. Sociodemographic Characteristics

Adolescents’ demographic profiles were assessed using four survey items, namely gender, residing region, living with parents, and parents’ working status (Table 1). Based on the Internet addiction test cut-off scores, 455 (27.9%) were identified as normal Internet users, 1067 (65.5%) belonged to the moderate-addiction or addiction-risk group, and 106 (6.5%) belonged to the severe addiction group. The group differences according to sociodemographic characteristics showed that 6.6% male and 6.4% female students were in the addiction group, and 66.7% male and 64.3% female students were deemed at risk for addiction (moderate addiction). There were no significant differences between three groups in regard to sociodemographic characteristics.

### 3.2. Internet Use Behaviors

Table 2 summarizes Internet use behaviors between normal use, moderate addiction, and severe addiction groups. About 74% of students had started using the Internet in grade school, and 19.7% had started using it while of preschool age. Of those who started using the Internet as early as before preschool, 9.4% were identified as addicted, compared to 10.0% and 5.4% who started using the Internet at preschool age and grade-school age, respectively. These group differences were statistically significant. More adolescents in the normal-use group reported a later age of first Internet use. Of the normal-use group, 81.3% (*n* = 370) started using the Internet in grade school (vs. 71.8% and 61.3% for the moderate addiction and addiction groups, respectively) (% not shown in Table 2). When students were asked to choose what Internet content they used regularly, the most frequently reported answers were webtoons, movies, or television (91.8%) and Social Networking Service or messenger (91.5%), followed by games (82.1%). Significant group differences were found for games and web surfing, with a higher percentage in the moderate addiction and severe addiction groups compared to the normal-use group.

### 3.3. Relationship between Psychological, Family/School, the Internet, and Environmental Characteristics and Internet Addiction

Table 3 summarizes the group differences in domains based on the public health model, namely psychological features, family/school factors, environmental factors, and Internet characteristics. For each domain, we focused on the variables that previous research had indicated were predictors of Internet addiction. One-way analysis of variance between groups was applied to examine differences between the normal use, moderate addiction, and severe addiction groups, followed by Tukey’s post-hoc test. Normality and homogeneity of variance assumptions were assessed. We first tested the normality through Shapiro–Wilk test and examined skewness and kurtosis. Shapiro–Wilk test rejected the null hypothesis and the absolute value for skewness and kurtosis did not exceed 2, which assumes a normal distribution of the variables. Levene’s test was performed to assess the homogeneity of variance and the results of the Games–Howell analysis were presented as a post hoc test for variables that do not assume equal variance. In order to confirm the differences among these groups, effect size was suggested. When adolescents in three groups were compared, the severe addiction group consistently had the highest level in all psychological features followed by the moderate addiction group and the normal use group. The differences were significant at the *p* < 0.001 level, although the effect size ranged from 0.01 to 0.24.

A comparison of family and school factors between the three groups showed a similar pattern. Students in the normal use group showed a significantly higher level of family cohesion, relationship with teachers, relationship with peers, attitude toward academic activities, and school participation. All differences were statistically significant.

The characteristics of the Internet content to which adolescents are drawn were compared. Mean scores in interpersonal relationship, anonymity, and entertainment characteristics were highest in the severe addiction group, followed by the moderate addiction group and then the normal use group, with the differences being statistically significant (*p* < 0.001).

Lastly, three environmental factors were compared between normal use, moderate addiction, and severe addiction groups, namely accessibility to PC cafés, exposure to Internet advertisements, and acceptability of Internet game advertisements. The mean score between the three groups was statistically significant (*p* < 0.001). Adolescents with addiction reported higher levels of accessibility to PC cafés, exposure to Internet ads, and acceptability of Internet game ads compared to the two other groups.

### 3.4. Factors Influencing Internet Addiction

Logistic regression was performed to determine the effect of variables in psychological, parent/school, environmental, and agent domains on the likelihood of having Internet addiction (Table 4). The severe addiction group was tested against the rest of the sample (moderate addiction and normal use groups) in order to determine the risk factors for the higher degree of Internet addiction. The logistic regression model was statistically significant (*χ*^2^ (26) = 231.29, *p* < 0.001), explaining 38.0% of the variance in Internet addiction among adolescents. Controlling for demographic and Internet use characteristics (gender, region, living with parents, age at first exposure to the Internet, etc.), six variables were found to increase the likelihood of Internet addiction. Among psychological factors, impulsiveness (Wald = 36.14, *p* < 0.001, Exp (B) = 1.277) and aggression (Wald = 4.42, *p* < 0.05, Exp (B) = 1.077) were identified as increasing the risk of Internet addiction. Among perceived Internet characteristics, a high perception of the Internet’s potential to build relationships (Wald = 12.17, *p* < 0.001, Exp (B) = 1.186) and anonymity (Wald = 8.80, *p* < 0.01, Exp (B) = 1.150) increased the risk of Internet addiction. Family and school factors were not significant predictors of Internet addiction. Two environmental factors, namely accessibility to PC cafés (Wald = 5.58, *p* < 0.05, Exp (B) = 1.116) and exposure to Internet game ads (Wald = 3.91, *p* < 0.05, Exp (B) = 1.26) were significant predictors of Internet addiction. Adolescents with higher accessibility to PC cafés and increased exposure to Internet game ads were respectively 1.12 and 1.26 times more likely to become addicted to the Internet.

## 4. Discussion

The present study investigated the predictors of Internet addiction based on the public health model using a representative sample of middle-school students in two major regions in Korea. More than half (72%) of Korean Adolescents in the study were categorized as moderate and severe addiction group, indicating that Internet addiction is a public health concern. Applying the public health model, several factors were found to be related to Internet addiction.

In regard to psychological factors, depressive symptoms, impulsivity, and aggressiveness were consistently higher among the addiction and moderate addiction groups compared to the normal use group. This finding is consistent with previous research that discussed the correlation between depression [18,28], impulsivity [23,31,65,66,67,68], aggression [22,27], and Internet addiction among young people. However, when other domains of the public health model were introduced, impulsivity and aggressiveness were found to predict Internet addiction. These associations are not surprising and are in line with extensive research on addictive behaviors, as cited in the introduction. Impulsivity is related to craving and dysfunctional coping with affective mood state, thereby increasing the risk of addictive behavior [67,68]. Impulsivity is one of the key symptoms of ADHD. The high comorbidity between ADHD and Internet addiction may be mediated by impulsivity [69]. With respect to aggressiveness, results are also consistent with past research showing that individuals with a high level of aggression also had a higher tendency toward Internet addiction [22,27]. However, since the study design is cross-sectional, it is difficult to determine whether the two traits were significant predictors for Internet addiction or the result of addiction. That is, impulsivity and aggressiveness may result from excessive Internet use and related problems. Future investigations are required to conclude whether impulsivity or aggressiveness result from, or are significant risk factors for, Internet addiction. Interestingly, the influence of depression became non-significant. One possible explanation could be that within the public health framework, depression does not affect one’s likelihood for Internet addiction as much as characteristics of the Internet or environmental factors.

This is similar to what we found regarding family and school-related variables. Findings from the bivariate analysis showed that adolescents who used the Internet without problems had a higher level of family cohesion and a more positive attitude toward academic activities compared to the addicted group. Previous studies also indicated that family factors were predictors of Internet addiction. For instance, for Italian adolescents, family members’ affective involvement has found to be associated with lower levels of Internet addiction [70]. Another study confirmed that inter-parental conflict predicted the incidence of Internet addiction among Taiwanese adolescents [35]. However, when other domains of the public health model were controlled for, the influence of family and school related variables became non-significant. Again, a possible explanation for this discrepancy may be related to the importance of environmental variables. Although the family and school environment is an important part of their life, larger environmental context related to the Internet may be more crucial in the development of Internet addiction among young people. Adolescents who experience lower family cohesion or find school life stressful may find refuge in nearby PC cafés where they can indulge themselves in games and Internet activities without distraction. This may be especially true for Korean youths, who are constantly under pressure for academic performance both in the family and at school.

Examination of the Internet’s characteristics in relation to addiction showed that the addicted group reported a higher level of entertainment (pleasure), anonymity, and interpersonal relationship factors. When other factors of the public health model were controlled, interpersonal relationships and anonymity were found to be associated with Internet addiction. Adolescents who perceived the Internet as a platform for socialization or building relationships were more likely to indulge in Internet use. This may indicate that the Internet is particularly compelling for those who otherwise find it hard to socialize. This result is in line with Chen and Kim’s [40] study that found the relationship building property to be positively related to problematic use of social network sites. Those who believe that acting freely without revealing their identity is an attractive aspect of the Internet were at increased risk for addiction. This may be similar to the discussion of having a fluid identity in online life as one of the seductive properties of the Internet [41]. These findings indicate that the characteristics of the Internet can affect adolescents’ Internet use behavior. To date, studies examining the influence of the Internet characteristics on Internet addiction within a public health framework are quite limited. More studies are needed to better understand the influence of traits of the Internet on Internet addiction.

Finally, our study results indicated that environmental factors are good predictors of Internet addiction in adolescents. The addicted adolescents reported higher accessibility to PC cafés and greater exposure to Internet game advertising. This held true after controlling for all other domains, namely individual, family and school, and Internet factors. PC café accessibility refers to the physical spaces in which the Internet can be used. Our findings support previous studies that have verified the effect of easy access to PC cafés on Internet addiction among Korean adolescents [47,60]. Availability and accessibility are well documented in substance abuse literature as risk factors for addictive behavior [48,49,71], and the current study’s results are consistent with the existing literature [72]. In addition, the influence of exposure to advertising is consistent with previous studies [73,74]. Our study revealed that adolescents who have more exposure to Internet game advertising and those who are more accepting of advertisements are more likely to become addicted. As a major public health policy, the World Health Organization emphasized regulating the availability and marketing of alcohol to prevent drinking-related problems [75]. We believe that the same can be applied to Internet addiction. For example, regulating the density of PC cafés and mandatory staff training to recognize and cope with excessive users can be suggested. Over the past ten years, there has been a tremendous increase in online game marketing in Korea. Despite this rapid growth, there are no regulations regarding the content of advertisements (e.g., violence, adult materials, etc.) or advertising models [76]. Adolescents are frequently exposed to Internet game ads with inappropriate and violent content. In addition, online game advertisements that feature popular actors are increasing significantly, which will likely attract young people. The current study results speak to the need for public policy that considers the effects of these environmental factors.

Most studies on Internet addiction have focused on individual factors, and even those that considered environmental influences were likely to examine the proximal environment such as familial and peer influence. One strength of this study is that it examined the broader environment such as accessibility to PC cafés and the exposure to advertisements that adolescents experienced in their community, which are common in Korea and some Asian countries. The findings confirmed the importance of the environmental influence on adolescents’ Internet addiction. At the same time, because PC cafés are unique to only some countries, the findings are applicable to the Korean context and few other cultures. When applying the model, researchers need to explore unique environmental features of the culture. Another limitation of the study is related to the definition of Internet characteristics. We included seductive properties of the Internet as the agent factor in the public health model. However, these properties may reflect the individual’s perception rather than the Internet itself. More research and further corroboration is needed to determine the characteristics of the Internet as the agent in relation to addiction. Since this study design was cross-sectional and could not illustrate a causal relationship, its results should be interpreted cautiously. There exists a possibility that some variables found to be influential such as impulsivity, aggressiveness, and the acceptability of online game ads may have been the result of Internet addiction. Further studies should examine whether these are risk factors for Internet addiction, the result of Internet addiction, or both. Despite its limitations, this study fills the gap in addiction literature by attempting to explain adolescents’ Internet addiction from a public health perspective.

## 5. Conclusions

Our study revealed several risk factors in the individual, Internet, and environmental domains. Although misuse of the Internet may be an individual’s choice, the current study speaks to the role of other factors such as the characteristics of the Internet and the environment in which individuals make their choice. Since these environmental factors are related to the profit-seeking of related industries, prevention efforts require a social policy approach. That is, strategies should target both changing individuals’ behaviors and setting the public health goal of reducing harmful Internet use at the country level. These may include regulations on PC café opening hours for adolescents and restrictions on the promotion and sponsorship of online games in youth activities. In 2019, the World Health Organization announced the inclusion of internet gaming disorder in the International Classification of Diaseses-11, and identified it as a health problem that requires a public health initiative. Further research is needed on intervention policies that specifically target individual and environmental risks based on the public health model. Findings of the current study may help in the planning of suitable strategies geared toward adolescent health.

## Figures and Tables

**Table 1:** Sociodemographic characteristics (*N* = 1628).

	*n* (%)/mean	Internet Addiction			*χ* ^2^
		Normal Use (*n* = 455)	Moderate Addiction (*n* = 1067)	Severe Addiction (*n* = 106)	
Gender					1.406 (df = 2) *p* = 0.495
Male	847 (52.0)	226 (26.7)	565 (66.7)	56 (6.6)	
Female	781 (48.0)	229 (29.3)	502 (64.3)	50 (6.4)	
Region					1.552 (df = 2) *p* = 0.460
Seoul	698 (42.9)	187 (26.8)	469 (67.2)	42 (6.0)	
Gyeonggi	930 (57.1)	268 (28.8)	598 (64.3)	64 (6.9)	
Living with parents					7.019 (df = 6) *p* = 0.319
Both	1496 (91.9)	415 (27.7)	987 (66.0)	94 (6.3)	
Father	41 (2.5)	17 (41.5)	21 (51.2)	3 (7.3)	
Mother	82 (5.0)	22 (26.8)	52 (63.4)	8 (9.8)	
Other	9 (0.6)	1 (11.1)	7 (77.8)	1 (11.1)	
Both parents working					0.796 (df = 2) *p* = 0.672
Yes	1075 (66.0)	304 (28.3)	705 (65.6)	66 (6.1)	
No	553 (34.0)	151 (27.3)	362 (65.5)	40 (7.2)	

**Table 2:** Internet use behavior (*N* = 1628).

	Totaln (%)	Internet Addiction (n, %)			*χ* ^2^	ω
		Normal Use (*n* = 455)	Moderate Addiction (*n* = 1067)	Severe Addiction (*n* = 106)		
Age of first Internet use					36.060 *** (df = 6)	0.15
Before preschool	53 (3.2)	12 (22.7)	36 (67.9)	5 (9.4)		
Preschool age	320 (19.7)	52 (16.2)	236 (73.8)	32 (10.0)		
Grade-school age	1201 (73.8)	370 (30.8)	766 (63.8)	65 (5.4)		
Other	54 (3.3)	21 (38.9)	29 (53.7)	4 (7.4)		
Internet contents						
Game	1337 (82.1)	339 (74.5)	913 (85.6)	85 (80.2)	26.877 *** (df = 2)	0.13
Web toon/movie/TV	1495 (91.8)	409 (89.9)	990 (92.8)	96 (90.6)	3.801 (df = 2)	
Adult materials	168 (10.3)	39 (8.6)	116 (10.9)	13 (12.3)	2.287 (df = 2)	
SNS, messenger	1489 (91.5)	409 (89.9)	985 (92.3)	95 (89.6)	2.893 (df = 2)	
Web surfing	860 (52.8)	214 (47.0)	576 (54.0)	70 (66.0)	14.125 ** (df = 2)	0.09

Note: Multiple answers were allowed for Internet contents. ** *p* < 0.01; *** *p* < 0.001. Effect size (ω) = 0.10 (small), 0.30 (medium), 0.50 (large) [64].

**Table 3:** Relationship between psychological, family/school, Internet, and environmental characteristics, and Internet addiction (*N* = 1628).

	Min	Max	Mean	SD	Internet Addiction Mean (SD)			F	$\eta^{2}$
					Normal (a)	Moderate Addiction (b)	Severe Addiction (c)		
Psychological factors									
Impulsivity	8	32	15.6	4.6	12.2 (4.2)	16.6 (4.0)	20.2 (4.1)	259.7 *** a < b < c (*df* = 2)	0.24
Depression	8	32	13.0	4.6	10.5 (3.7)	13.8 (4.3)	16.6 (5.0)	134.4 *** a < b < c (*df* = 2)	0.14
Aggressiveness	8	32	13.8	5.1	10.8 (4.1)	14.7 (4.7)	18.4 (5.2)	171.4 *** a < b < c (*df* = 2)	0.18
Family/school factors									
Family cohesion	10	50	37.3	8.2	38.8 (9.8)	36.7 (7.4)	36.2 (8.0)	11.5 *** a > b, c (*df* = 2)	0.01
Relationship with teachers	3	15	10.9	2.6	11.5 (3.0)	10.6 (2.4)	10.6 (2.5)	20.1 *** a > b, c (*df* = 2)	0.02
Peer relationships	3	15	11.6	2.6	12.1 (2.8)	11.3 (2.5)	11.5 (2.5)	15.3 *** a > b (*df* = 2)	0.02
Academic activities	5	25	16.8	3.1	17.6 (3.7)	16.5 (2.8)	17.1 (2.9)	20.2 *** a > b (*df* = 2)	0.02
School participation	3	15	10.5	2.1	11.0 (2.4)	10.2 (2.0)	10.8 (2.2)	25.8 *** a > b, b>c (*df* = 2)	0.03
Perceived Internet characteristics									
Relationship	4	20	10.1	4.1	8.0 (4.0)	10.8 (3.7)	13.2 (4.0)	125.8 *** a < b < c (*df* = 2)	0.14
Anonymity	4	20	9.2	4.0	6.6 (3.5)	10.0 (3.6)	12.5 (4.1)	189.8 *** a < b < c (*df* = 2)	0.19
Entertainment	5	25	13.2	5.5	10.1 (5.3)	14.3 (4.9)	16.2 (6.0)	122.0 *** a < b < c (*df* = 2)	0.13
Environmental factors									
Accessibility of PC cafés	3	15	10.2	3.3	9.4 (3.6)	10.4 (3.2)	11.4 (3.1)	21.8 *** a < b < c (*df* = 2)	0.03
Exposure to Internet game ads	2	8	5.9	1.4	5.6 (1.6)	6.0 (1.3)	6.5 (1.3)	26.5 *** a < b < c (*df* = 2)	0.03
Acceptability of Internet game ads	1	10	5.6	3.0	5.2 (3.0)	5.7 (3.0)	6.4 (3.1)	8.0 *** a < b < c (*df* = 2)	0.01

Note: *** *p* < 0.001. Effect size ($\eta^{2}$) = 0.01 (small), 0.06 (medium), 0.14 (large) [64].

**Table 4:** Logistic regression analysis of factors influencing Internet addiction.

	*B*	*S. E.*	*Wald*	*OR*	*Exp* (*B*) 95% *CI*	
					Lower	Upper
Psychological factors						
Impulsivity	0.244	0.041	36.140	1.277 ***	1.179	1.382
Depression	0.024	0.036	0.464	1.025	0.956	1.098
Aggressiveness	0.075	0.035	4.417	1.077 *	1.005	1.155
Family/school factors						
Family cohesion	0.026	0.020	1.622	1.026	0.986	1.067
Relationship with teachers	−0.058	0.067	0.746	0.943	0.827	1.077
Peer relationship	0.001	0.060	0.001	1.001	0.891	1.125
Academic activities	0.070	0.060	1.336	1.072	0.953	1.207
School participation	−0.031	0.079	0.155	0.970	0.831	1.131
Internet characteristics						
Relationship	0.170	0.049	12.174	1.186 ***	1.078	1.305
Anonymity	0.140	0.047	8.796	1.150 **	1.049	1.261
Entertainment	−0.014	.032	0.186	0.986	0.926	1.050
Environmental factors						
Accessibility to PC café	0.110	0.047	5.581	1.116 *	1.019	1.223
Exposure to Internet game ads	0.230	0.116	3.914	1.259 *	1.002	1.581
Acceptability of Internet game ads	−0.017	0.045	0.135	0.984	0.900	1.075
Gender (Male = 1)	−0.418	0.309	1.837	0.658	0.360	1.205
Region (Seoul = 1)	−0.507	0.266	3.616	0.603	0.358	1.016
Live with parents	−0.472	0.393	1.445	0.624	0.289	1.347
Both parents working	−0.237	0.267	0.784	0.789	0.468	1.332
Age of first Internet use 1	0.259	0.875	0.088	1.296	0.233	7.208
Age of first Internet use 2	−0.285	0.696	0.168	0.752	0.192	2.942
Age of first Internet use 3	−0.651	0.669	0.946	0.522	0.140	1.936
Game	−1.097	0.385	8.105	0.334 **	0.157	0.710
Web toon/movie/TV	−0.388	0.485	0.642	0.678	0.262	1.753
Adult materials	−0.171	0.422	0.165	0.843	0.368	1.927
SNS/messenger	−0.606	0.440	1.899	0.546	0.230	1.292
Web surfing	0.538	0.282	3.653	1.713	0.986	2.976
*χ* ^2^	231.287 (*df* = 26) ***					
*−2LL*	472.806					
*Nagelkerke R* ^2^	0.380					

Note: Age of Internet use was dummy coded as the following: Age of first Internet use 1 = before preschool; Age of first Internet use 2 = preschool age; Age of first Internet use 3 = grade school; *B* = coefficient; *SE* = standard error of the coefficient; *OR* = odds ratio; *CI* = confidence interval; * *p* < 0.05; ** *p* < 0.01; *** *p* < 0.001.
